# Toward the automated generation of genome-scale metabolic networks in the SEED

**DOI:** 10.1186/1471-2105-8-139

**Published:** 2007-04-26

**Authors:** Matthew DeJongh, Kevin Formsma, Paul Boillot, John Gould, Matthew Rycenga, Aaron Best

**Affiliations:** 1 Department of Computer Science, Hope College, 27 Graves Place, Holland, MI, USA; 2 Department of Biology, Hope College, 35 E. 12^th ^St., Holland, MI, USA; 3 Department of Chemistry, Hope College, 35 E. 12^th ^St., Holland, MI, USA; 4 Department of Chemistry, University of Washington, Box # 351700, Seattle, WA, USA

## Abstract

**Background:**

Current methods for the automated generation of genome-scale metabolic networks focus on genome annotation and preliminary biochemical reaction network assembly, but do not adequately address the process of identifying and filling gaps in the reaction network, and verifying that the network is suitable for systems level analysis. Thus, current methods are only sufficient for generating draft-quality networks, and refinement of the reaction network is still largely a manual, labor-intensive process.

**Results:**

We have developed a method for generating genome-scale metabolic networks that produces substantially complete reaction networks, suitable for systems level analysis. Our method partitions the reaction space of central and intermediary metabolism into discrete, interconnected components that can be assembled and verified in isolation from each other, and then integrated and verified at the level of their interconnectivity. We have developed a database of components that are common across organisms, and have created tools for automatically assembling appropriate components for a particular organism based on the metabolic pathways encoded in the organism's genome. This focuses manual efforts on that portion of an organism's metabolism that is not yet represented in the database. We have demonstrated the efficacy of our method by reverse-engineering and automatically regenerating the reaction network from a published genome-scale metabolic model for *Staphylococcus aureus*. Additionally, we have verified that our method capitalizes on the database of common reaction network components created for *S. aureus*, by using these components to generate substantially complete reconstructions of the reaction networks from three other published metabolic models (*Escherichia coli*, *Helicobacter pylori*, and *Lactococcus lactis*). We have implemented our tools and database within the SEED, an open-source software environment for comparative genome annotation and analysis.

**Conclusion:**

Our method sets the stage for the automated generation of substantially complete metabolic networks for over 400 complete genome sequences currently in the SEED. With each genome that is processed using our tools, the database of common components grows to cover more of the diversity of metabolic pathways. This increases the likelihood that components of reaction networks for subsequently processed genomes can be retrieved from the database, rather than assembled and verified manually.

## Background

The availability of hundreds of sequenced genomes has ushered in a new era in biology, allowing the study of cellular life at a systems level. One approach to systems level understanding of cellular life is *in silico *modeling of an organism's metabolic capabilities, as determined by the complement of genes in its genome [1]. For example, flux balance analysis is a widely used modeling technique which enables the prediction of metabolic phenotypes based on the enzymes encoded in an organism's genome [2-5]. The model consists in part of a network of biochemical reactions that represent the activity of these enzymes. The usefulness of the model is subject to the accuracy of the reaction network upon which it is based: the reaction network should be *complete*, fully covering the metabolic capabilities that are to be modeled, *coherent*, containing no gaps or dead ends, and *correct*, faithfully representing the metabolic phenotype of the organism. The accuracy of the reaction network can be tested by comparing the predictions of the model with the known metabolic phenotype of the organism under (pseudo) steady-state conditions.

A major challenge of genome-scale metabolic modeling is to reconstruct an accurate reaction network directly from an annotated genome [6]. There are four steps required to generate an accurate reaction network for an organism:

*1. Annotating *the genome to identify the enzymes encoded by particular genes.

*2. Assembling *the network of reactions that correspond to these enzymes.

*3. Verifying *the completeness and coherence of the assembled reaction network.

*4. Testing *the correctness of the assembled reaction network using a modeling technique such as flux balance analysis.

**Figure 1:**
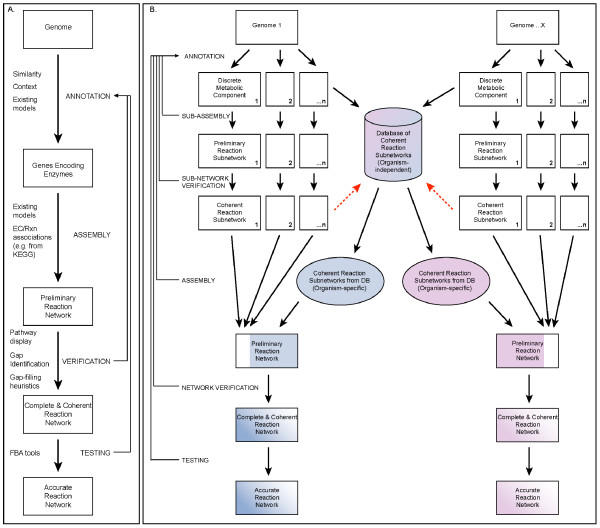
**Overview of genome-scale metabolic reconstruction**. (A) A typical process for generating genome-scale metabolic reaction networks. Arrows correspond to the four steps of the reconstruction process – Annotation, Assembly, Verification, and Testing. The types of information and methods used during each step are indicated to the left of the arrows in the diagram. Steps in the process that feed back to refine the initial annotation are depicted as arrows on the right side of the diagram to illustrate the iterative nature of the process. (B) Our approach to genome-scale metabolic reconstruction generates an organism-independent database of coherent reaction subnetworks for discrete components of central and intermediary metabolism. This database is used to generate an organism-specific set of coherent reaction subnetworks based on the genome annotation. This set is then combined with reaction subnetworks constructed for metabolic components not represented in the database, resulting in a preliminary reaction network. Each new metabolic reconstruction adds coherent reaction subnetworks specific to new metabolic components to the database (depicted as dashed, red arrows).

These steps represent an iterative process [7] (Fig. 1A). For example, the verification step may reveal gaps in the assembled network, which in turn may reveal incorrect or missing gene annotations. This interplay between annotation, assembly, verification, and testing is a valuable process, as it results in the refinement of both the genome annotation and the reaction network. Currently, refinement of the genome annotation and reaction network is largely a manual, labor-intensive process [8]. Work to automate the generation of genome-scale metabolic networks has primarily focused on preliminary annotation and assembly. Automated annotation procedures rely heavily on sequence similarity searching of existing genome and functional motif databases. This technique inevitably leaves gaps and dead ends when the reaction network is assembled. Recent efforts to address this shortcoming take into account genome and metabolic pathway context to attempt to identify missing genes that fill these gaps [9-11]. One approach to automating reaction network assembly [12-15] is to populate the network directly from information about enzymes included in the genome annotation. This is usually done by consulting databases that encode relationships between EC numbers and specific reactions (*e.g.*, KEGG [16], MetaCyc [17]). The downside of this approach is that a given EC number may represent a set of related reactions, not all of which are necessarily catalyzed by every organism's corresponding gene product. A complementary approach is to incorporate associations between genes and specific reactions from existing genome-scale metabolic models in the annotation process [18]. In each case, the assembled networks serve as starting points, requiring verification and refinement before they are suitable for modeling.

Few techniques have been proposed for automating the verification of an assembled reaction network (Fig. 1A). Segrè *et al. *[19] describe an algorithm that searches the reaction network to identify essential intermediate metabolites that cannot be synthesized by other reactions in the network. Arakawa *et al. *[12] apply a gap-filling heuristic to connect segments of pathways that represent consecutive reactions, and flag the filled gap for subsequent manual curation. Other systems require the user to identify gaps, and assist in this process by providing tools to visualize genome annotations in the context of metabolic pathway maps [13-15]. This information can be used to improve the accuracy of the reaction network, by feeding back into the annotation and assembly steps for iterative refinement.

Only a handful of metabolic reconstructions suitable for modeling have been published to date [20-29]. With the completion of the 1000^th ^microbial genome predicted for late 2007 [11], there is an urgent need for more substantial automation of the process of generating complete and coherent reaction networks suitable for testing at the systems level.

We have developed an integrated suite of tools that supports iterative annotation, assembly, verification and refinement of genome-scale metabolic reaction networks. Our approach is based on partitioning central and intermediary metabolism into discrete, interconnected components that are shared across organisms (Fig. 1B). Each of these components represents a subnetwork of reactions within an overall metabolic reaction network. In our approach, each reaction subnetwork is assembled and verified independently, and stored in a database so that it can be used for multiple organisms. An organism-specific reaction network is assembled by retrieving appropriate reaction subnetworks from the database and verifying their coherence at the level of their connectivity. Any effort required to assemble and verify reaction subnetworks for components that are not already in the database has a cumulative effect, because those reaction subnetworks subsequently can be stored in the database and used for other organisms.

**Figure 2:**
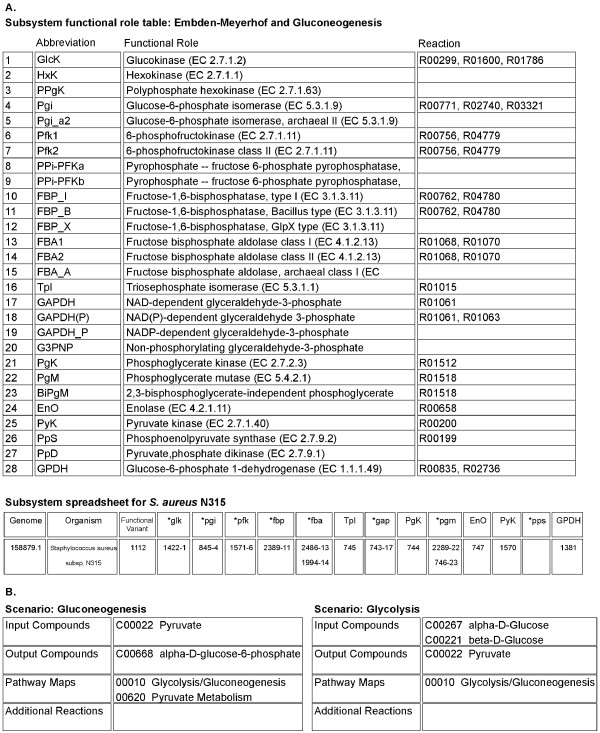
**Subsystem functional role table, spreadsheet and scenarios in the SEED**. (A) The Functional Role table and Spreadsheet for the *Embden-Meyerhof and Gluconeogenesis *subsystem are depicted. The Functional Role table includes short abbreviations, functional role names and associated KEGG reaction identifiers. The Spreadsheet contains columns corresponding to functional roles and rows corresponding to specific genomes (only *S. aureus *subsp. N315 shown). The cells of the spreadsheet contain gene identifiers that have been annotated with the specified functional role. A column heading containing an asterisk (*) indicates that several related functional roles are grouped together in a column; subscripts after gene identifiers indicate which functional role the organism implements (e.g., 1571-6 for *pfk). (B) We have encoded *scenarios *in subsystems that represent segments of metabolic pathways. The elements of each scenario are input compounds, output compounds, the KEGG pathway maps that provide context for the scenario, and additional reactions.

We have implemented our approach within the SEED, a community-based genome annotation and analysis environment [30]. The SEED implements a cross-organismal approach to genome annotation called the *subsystems *approach [11], in which experts in particular biological processes focus on annotating genes involved in those biological processes across the complete collection of sequenced genomes. The SEED defines a *subsystem *as a set of related functions of gene products (termed *functional roles*), and a set of relations between functional roles and the genes that encode the corresponding products in particular organisms (termed the *spreadsheet*). A subsystem can represent any relationship between functional roles, such as the set of enzymes that make up a metabolic pathway (*e.g.*, the Embden-Meyerhof pathway, Fig. 2A). Subsystems reveal variations on how particular biological processes are implemented by various organisms. For example, in a subsystem describing the degradation of histidine, three distinct forms of degradation are used by different groups of organisms. The distinct sets of functional roles that represent these variations within a subsystem are termed *functional variants*; for every organism in the spreadsheet, the particular functional variant that it corresponds to is identified. The SEED already contains subsystems that cover the diversity of central and intermediary metabolism across many organisms. The functional roles in these subsystems include the enzymes that make up the metabolic pathways represented by the subsystems. The SEED provides the capability of associating reactions with these functional roles in an organism-independent manner. These fundamental properties of subsystems – grouping of related functional roles, association of reactions with functional roles, annotation of specific genes to functional roles across many genomes, and identification of functional variants – provide the foundation for our approach to constructing metabolic reaction networks for organisms in the SEED.

The SEED provides tools for the automated identification of genes, annotation of genes to functional roles and propagation of annotated genes into subsystems, as well as tools for the manual curation of gene annotations [11]. We have extended the SEED to enable the assembly and verification of metabolic reaction networks, by creating tools for the purpose of (1) curating associations between functional roles and reactions in subsystems that represent metabolic pathways; (2) assembling and verifying the coherence of reaction subnetworks in these subsystems; (3) assembling and verifying the coherence of connected reaction subnetworks across related subsystems; and (4) assembling and verifying the coherence and completeness of an organism-specific reaction network (Table 1). In the following section, we elaborate on our approach, our extensions to the SEED, and the tools we have written for reaction network construction.

**Table 1:** Software tools developed for genome-scale metabolic reconstruction

**Steps**	**Purpose of Tools**	**Implementation in the SEED**	**Database Contributions**
ANNOTATION		The SEED already provides tools for annotation, based on similarity searching and context-based methods	The SEED already provides a database of high-quality genome annotations organized into subsystems (see [11])

SUB-ASSEMBLY AND SUB-NETWORK VERIFICATION	Curating associations between functional roles and reactions in a particular metabolic context	Reverse-engineering of published genome-scale metabolic models; analysis of gene-reaction associations in the KEGG database; integrated display of KEGG pathway maps in subsystems, highlighting functional roles and associated reactions	Associations between functional roles and KEGG reactions in subsystems
	
	Assembling and verifying the coherence of reaction subnetworks in subsystems	Petri net representation of KEGG reactions; encoded scenarios in subsystems; finding paths through reaction subnetworks from scenario inputs to scenario outputs	Reuseable coherent reaction subnetworks in subsystems
	
	Assembling and verifying the coherence of connected reaction subnetworks across subsystems	Connections between scenarios in different subsystems; finding paths through connected scenarios, from overall inputs to overall outputs	List of curated subsystems with coherent reaction subnetworks for functional variants that interconnect to cover central and intermediary metabolic pathways

ASSEMBLY AND NETWORK VERIFICATION	Assembling and verifying the coherence and completeness of an organism-specific reaction network	Identifying gaps in the reaction network, by cross-checking inputs and outputs for all paths through implemented scenarios, and checking for paths from minimal substrates to biomass compounds; creating files for FluxAnalyzer [36]	Organism-specific complete and coherent reaction networks for central and intermediary metabolism

## Approach

### Extending subsystems to represent reaction subnetworks

As mentioned above, subsystems within the SEED already have the capability of associating reaction information with functional roles. The reaction information, when it is present, is specified by links to reaction identifiers in the KEGG database. Prior to our work, only a small percentage of functional roles in the SEED had reaction information associated with them. To address this shortcoming, we have reverse-engineered existing genome-scale metabolic models to determine the correspondence between the reactions in these models and functional roles in subsystems (see Methods).

We have created encodings of segments of metabolic pathways within subsystems, which we call *scenarios *(Fig. 2B). A scenario represents a set of connected reactions that convert a defined set of substrates (*scenario inputs*) to a defined set of products (*scenario outputs*). For example, the *Embden-Meyerhof and Gluconeogenesis *subsystem contains functional roles and associated reactions for the metabolic pathway of glycolysis. We have encoded a *Glycolysis *scenario within this subsystem, for which the inputs are alpha- and beta-D-glucose, and the output is pyruvate. The input and output compounds for scenarios in subsystems are encoded using KEGG compound identifiers (*e.g., C00267 *for alpha-D-glucose). A scenario additionally encodes KEGG pathway map identifiers that provide context for the set of connected reactions that convert the scenario inputs to the scenario outputs. The pathway maps contain information about reaction reversibilities, and identify the "main" compounds in each reaction (*e.g., *the molecules derived from the breakdown of glucose), as opposed to cofactors. A scenario does not explicitly encode this set of connected reactions – it is assembled dynamically from the reactions associated with functional roles in the subsystem, using a process described below to search for paths through the reactions from the scenario inputs to the scenario outputs. However, a scenario can specify additional reactions that are not associated with functional roles, yet play a part in the scenario (*e.g., *ionization reactions and spontaneous reactions that are not catalyzed by an enzyme).

**Figure 3:**
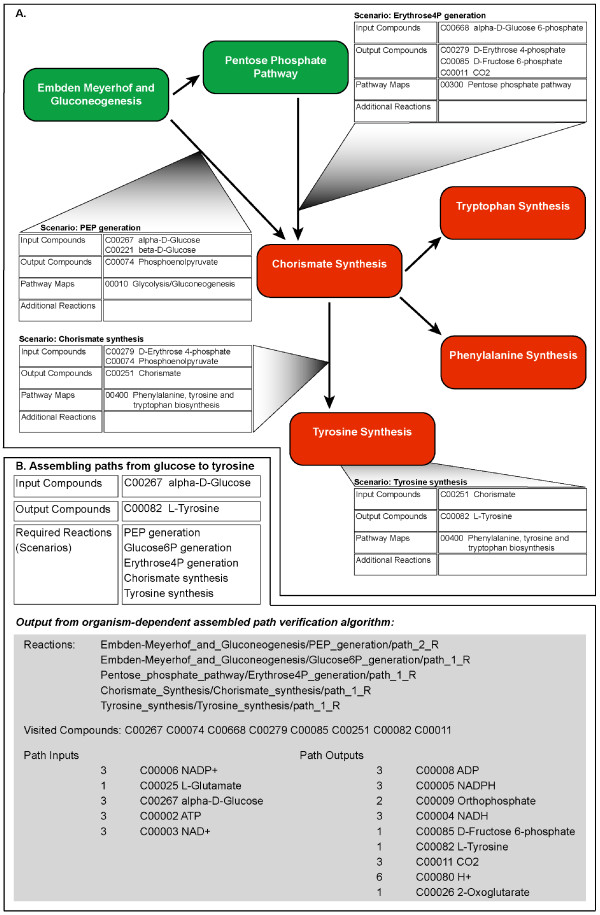
**Connected scenarios across multiple subsystems**. (A) Subsystems in the SEED are depicted as colored boxes and the connections between the subsystems are shown as arrows. Directionality of the arrows shows the flow of output compounds from scenarios in one subsystem that are used as input compounds for scenarios in another subsystem. Four connections between subsystems are expanded to display the scenario within one subsystem that is connected to a scenario in a different subsystem. In this case, a set of subsystems and scenarios is depicted that represents the conversion of glucose into the amino acid tyrosine. (B) We have developed a tool to find paths through connected scenarios across multiple subsystems. The table shows the initial input compound, target output compound and the necessary scenarios to complete the conversion. Each scenario is treated as a "higher-order" reaction by the tool. Output from the tool shows which scenarios were used, which main compounds were used, and the stoichiometry of the overall inputs and outputs necessary to convert the input compound to the output compound.

Scenario inputs and outputs can be used to identify points of connection between the reaction subnetworks represented by different subsystems. For example, the *Chorismate Synthesis *subsystem contains functional roles that represent the set of reactions that convert erythrose-4-phosphate and phosphoenolpyruvate into chorismate. Accordingly, we have added a *Chorismate synthesis *scenario to this subsystem. Because chorismate is a precursor for synthesizing tryptophan, phenylalanine and tyrosine, we have created scenarios within the three corresponding subsystems (*Tryptophan synthesis*, *Phenylalanine synthesis*, and *Tyrosine synthesis*), which specify chorismate as a scenario input, and the respective aromatic amino acid as a scenario output (Fig. 3A).

### Curating associations between functional roles and reactions in subsystems

The first step in ensuring that a subsystem contains a coherent subnetwork of reactions is to associate appropriate reactions with the functional roles in the subsystem. Functional roles that correspond to enzymes usually specify an EC number. As noted above, an EC number often designates a set of related reactions; however, a particular organism's gene product may not implement all of the reactions associated with a given EC number. In the SEED, a functional role can be associated with multiple subsystems, and a different set of reactions can be specified for the functional role in each of these subsystems. Thus an organism's gene product will only be associated with the reactions in a particular subsystem if the gene is linked to the associated functional role by that subsystem's spreadsheet. The metabolic pathways represented by each subsystem provide context for determining which reactions should be associated with a functional role in the subsystem. For example, EC 1.1.1.1 (alcohol dehydrogenase) is associated with 12 reactions in the KEGG database. These 12 reactions have a variety of substrates, including all primary and secondary alcohols. The KEGG entry for *E. coli K12 *gene *b1241 *[31] annotates it with EC 1.1.1.1, but does not identify its substrate specificity. The SEED entry for this gene includes annotation with the functional role "Alcohol dehydrogenase (EC 1.1.1.1)," and shows that it is present in several subsystems, including *Fermentations: Lactate*, for which the associated reaction is R00754 (substrate is ethanol), and *Glycerolipid and Glycerophospholipid metabolism*, for which the associated reaction is R01036 (substrate is glycerol).

To determine which reactions to associate with a functional role in a particular subsystem, we have integrated the display of KEGG pathway maps into the subsystem environment. KEGG pathway maps display metabolic pathways as graphs, where the nodes represent compounds and the edges represent reactions. Edges that represent enzymatically catalyzed reactions are drawn with a box containing the EC number corresponding to the enzyme. KEGG provides the capability of displaying maps with a particular set of edges highlighted, either by specifying the corresponding EC numbers or the corresponding reactions. We have developed a tool which searches for any KEGG map containing one or more of the EC numbers specified by the functional roles in the subsystem, and displays links to the maps in order of decreasing number of EC matches. For each map, two links are displayed: one that highlights the EC numbers that were matched, and one that highlights the reactions associated with functional roles in the subsystem that were matched. These displays are useful for visually determining the extent to which the functional roles and reactions in the subsystem form a connected and complete subnetwork. We use this tool to identify gaps in the reaction subnetwork, which we fill by adding functional roles to the subsystem corresponding to unmatched EC numbers, and by adding reaction associations to appropriate functional roles for unmatched reactions.

### Assembling and verifying reaction subnetworks in subsystems

**Figure 4:**
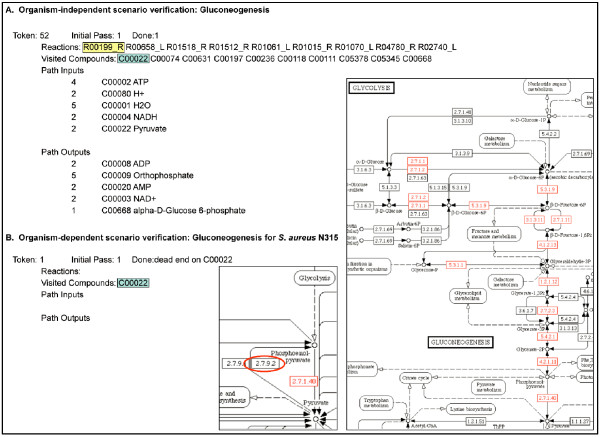
**Verification of pathway completeness using scenarios**. A Petri net algorithm is used to find all possible paths through a subsystem's reactions to complete a given scenario. This can be executed in an organism-independent or dependent manner. Output of the tool includes those reactions that make up the path completing the scenario, the KEGG compounds used, and the stoichiometry of the overall inputs and outputs of the scenario. Each reaction is appended with an R or an L to indicate the direction of the reaction used in the context of the pathway. (A) The first example shows the results of an organism-independent execution of the algorithm for the scenario *Gluconeogenesis *in the *Embden-Meyerhof and Gluconeogenesis *subsystem. (B) The second example shows the results of an organism-dependent execution of the algorithm for the same scenario. Here the results indicate that *S. aureus *does not contain a complete path in this subsystem for gluconeogenesis. The output indicates where the failure occurred, which is the first reaction in the pathway converting pyruvate (highlighted in blue) to phosphoenolpyruvate. The conversion is performed by reaction R00199_R (highlighted in yellow). The *Glycolysis and Gluconeogenesis *and *Pyruvate Metabolism *KEGG maps with reactions highlighted in an organism-dependent manner for *S. aureus *give a visual context for the results of the algorithm. The reaction R00199 (indicated by a red circle) is not highlighted on the KEGG map; consulting the subsystem spreadsheet reveals that *S. aureus *does not contain the gene encoding phosphoenolpyruvate synthase (EC 2.7.9.2) (*cf. *Fig. 2, role 26).

We have developed a tool that uses a Petri net [32-35] representation of KEGG reactions to find all possible paths from scenario inputs to scenario outputs through a given set of reactions. This tool makes use of reaction reversibilities and the distinction between "main" and "non-main" compounds in KEGG pathway maps to push "tokens" representing metabolites and cofactors through reactions from scenario inputs to scenario outputs. When the set of reactions forms a connected subnetwork, the tool reports all paths that were found, including the reactions that form each path, and the cumulative stoichiometry for all compounds that are inputs and outputs for each path (Fig. 4A). When gaps are present in the reaction subnetwork, the tokens halt on compounds that are not scenario outputs. These "dead ends" are reported and can be used to locate the gaps and identify reactions that are needed to fill them. By default, this tool uses all of the reactions associated with the subsystem's functional roles to find all possible paths through the reactions in an organism-independent manner. In conjunction with the integrated display of KEGG pathway maps, this is useful for ensuring that the reactions in the subsystem form a coherent subnetwork. In addition, this organism-independent use of the path-finding tool reveals the scope of potential functional variation encoded in a subsystem, as represented by the diversity of paths through the reaction subnetwork.

In order to determine which scenarios, if any, are implemented by a given organism, we filter the set of reactions used by the path-finding tool according to the functional roles annotated to the organism's genome in the subsystem's spreadsheet (Fig. 4B). Gaps in the organism-specific reaction subnetwork indicate that further genome annotation is required to determine whether genes responsible for the missing reactions are present. If these genes are identified and added to the spreadsheet, subsequent reaction subnetwork assembly and verification will result in a coherent organism-specific reaction subnetwork with paths through the scenario. In this case, the existence of paths through the scenario is ensured not only for this organism, but also for any other organism which corresponds to the same functional variant, *i.e., *whose genome is annotated with the same set of functional roles.

The reaction subnetworks encoded in subsystems form the database of reusable metabolic network components (Fig. 1B). By verifying that the reaction subnetwork in a given subsystem is coherent for all the scenarios implemented by a particular functional variant, we have effectively verified the coherence of the reaction subnetwork for any organism that corresponds to that functional variant. Thus, the task of assembling a coherent reaction subnetwork from the subsystem for a given organism is accomplished by determining the organism's corresponding functional variant in the spreadsheet, and assembling the reactions associated with the corresponding set of functional roles. Then the path-finding tool can be applied to determine which scenarios are implemented by that functional variant.

### Assembling and verifying connected reaction subnetworks

As described above, reaction subnetworks across related subsystems are connected by shared scenario inputs and outputs (Fig. 3A). We assemble connected reaction subnetworks by collecting all of the reactions associated with a specified list of subsystems. We use the same path-finding tool to search for paths through connected reaction subnetworks; however, instead of constructing the Petri net using KEGG reactions from the subsystems, we represent each possible path through a given scenario as a distinct reaction. These "higher-order" reactions have the path's input compounds as substrates and the path's output compounds as products, and use the cumulative stoichiometry that was previously determined by the path-finding tool. For example, the path through the *Gluconeogenesis *scenario shown in Fig. 4A can be represented as a higher-order reaction that converts 2 molecules of pyruvate (plus cofactors) to 1 molecule of alpha-D-Glucose 6-phosphate (plus cofactors). By matching the outputs of one higher-order reaction to the inputs of another, the path-finding tool pushes tokens from a set of overall input compounds to a set of overall output compounds. For example, the *PEP (phosphoenolpyruvate) generation *scenario in the *Embden-Meyerhof and Gluconeogenesis *subsystem and the *Erythrose4P (erythrose-4-phosphate) generation *scenario in the *Pentose Phosphate Pathway *subsystem generate the scenario inputs for the *Chorismate synthesis *scenario in the *Chorismate Synthesis *subsystem. The output of the *Chorismate synthesis *scenario in turn serves as input to the scenarios for the synthesis of the aromatic amino acids. The Petri net tool can be used to find paths through these scenarios that convert glucose to tryptophan, phenylalanine, and tyrosine on both an organism-independent and organism-specific basis (Fig. 3B). As before, dead end tokens signal gaps in the assembled network, which in this case represent missing scenarios.

### Assembling and verifying an organism-specific reaction network

To assemble an organism-specific reaction network, we collect all of the reactions associated with the organism's genome annotation in a specified list of subsystems. To verify that this reaction network is coherent and complete, we use our path-finding tool to determine which of the scenarios encoded in the subsystems are implemented by the organism. We cross-check the input and output compounds for all of these paths to determine which input compounds are not outputs for any of the paths, and which output compounds are not inputs for any of the paths. Ideally, these compounds correspond respectively to potential reaction network inputs (*e.g*., sugars that are transported into the cell by the organism) and outputs (*e.g., *amino acids that are synthesized by the organism for biomass growth). If any of these compounds represent intermediate steps in metabolism, the subsystems need to be checked for scenarios that produce or consume them accordingly, and the organism's annotation in these subsystems needs to be checked with respect to the functional roles necessary to implement the missing scenarios.

An organism-specific reaction network that is suitable for flux balance analysis must additionally specify transport and exchange reactions, a biomass equation, and a set of minimal substrates that can be used to generate the biomass compounds for the organism. Currently, these additional components must be supplied by hand for a given organism. We have written a tool that, given these supplied components and subsystem reactions, assembles them into a final reaction network, and outputs the network in a format suitable for loading into the FluxAnalyzer [36]. To verify the completeness of the final reaction network, the tool determines whether there is a path through the network from the set of minimal substrates to each biomass compound. This tool uses a breadth-first search algorithm (similar to the algorithm described in [19]), starting with the minimal substrates, to determine all the ways that paths through scenarios can be connected to form paths to the biomass compounds. It "borrows" compounds along the way, if necessary, to find complete paths through the scenarios, then reports on the paths it found and the compounds it had to borrow. Any borrowed compounds must be accounted for, either by adding more transport reactions or creating new scenarios to produce them. The final step is to load the network into the FluxAnalyzer, supply minimal substrates, and verify flux through the biomass reaction.

## Results

### Reverse-engineering and regenerating the *S. aureus *metabolic reaction network

**Figure 5:**
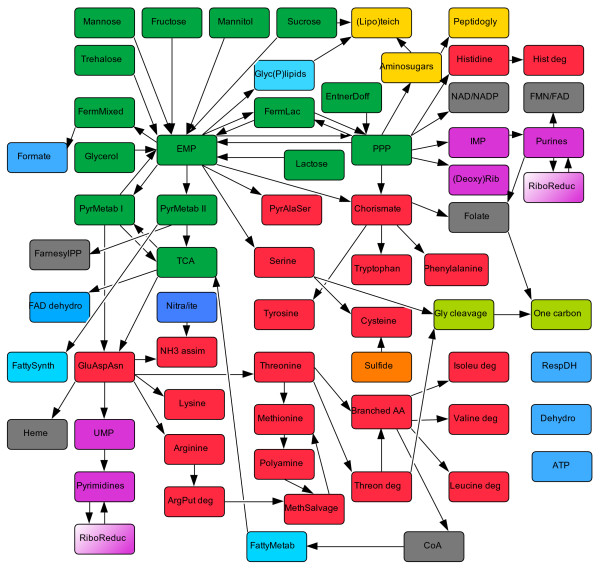
**Connections between subsystems in the SEED**. The network of interactions that occur among 65 subsystems in the SEED used to regenerate the *S. aureus *model. Each box represents a subsystem. Colors correspond to the categories of metabolism described by the 2003 International Union of Biochemistry and Molecular Biology Metabolic Pathways chart (Sigma-Aldrich, ). Carbohydrates, green; amino acids, red; lipids, light blue; purines and pyrimidines, purple; and vitamins, coenzymes and hormones, gray. Abbreviations within subsystem boxes are explained in Additional File 2.

In order to test the efficacy of our approach, we have reverse-engineered the reaction network from a published genome-scale metabolic model for *Staphylococcus aureus *N315 [20] into the SEED (see Methods). We have curated associations between reactions and functional roles in 65 subsystems and created a total of 133 interconnected scenarios, covering the major pathways for amino acid, carbohydrate, cell wall, lipid, nucleotide, vitamin and cofactor metabolism (Fig. 5). We have used the tools described above to assemble a complete and coherent metabolic reaction network for *S. aureus *N315 based solely on its genome annotation in these subsystems. We have verified that the reaction network is suitable for flux balance analysis, using the transport reactions and biomass reaction from the *iSB619 *model, exchange reactions for each transported compound, and the list of minimal substrates specified in supporting materials [37].

Table 2 documents the number and categories of reactions in the metabolic reaction network. The number of reactions automatically assembled from subsystems (518) is higher than the number of reactions from the original model that we mapped to functional roles (394). Many of these are accounted for by parallel reactions that process alternative carbohydrate forms (see Methods). Others are due to the presence of functional roles in the 65 curated subsystems that extend beyond the pathways represented by the model. For example, S-adenosyl-L-homocysteine (*ahcys*) is a dead end in the model, even though it is produced by the model as a by-product of methionine metabolism. Therefore the model contains an exchange reaction (*sink_ahcys*) to ensure that S-adenosyl-L-homocysteine does not pool and block flux through the rest of the model. The *Methionine metabolism *subsystem, on the other hand, contains functional roles that represent the recycling of S-adenosyl-L-homocysteine to adenine, D-ribose and L-homocysteine, which is a precursor to L-methionine. The associated reactions (R00194 and R01291) are included in our regenerated network, so the *sink_ahcys *exchange reaction is not needed.

**Table 2:** Regenerated *S. aureus *network

Total Reactions	812
***Reaction Categories***	

Reactions Automatically assembled from Subsystems	518
Reactions Added "As is" from *iSB619 *Model^a^	33
Conversion Reactions^b^	24
Transport Reactions from *iSB619 *Model	93
Exchange Reactions for Transported Compounds	144

^a^. Reactions in *iSB619 *model that could not be mapped to KEGG reactions (see Methods).

^b^. Reactions to convert model compound IDs to KEGG compound IDs.

### The cumulative effect of the database of coherent reaction subnetworks

Although our initial efforts have focused on generating the metabolic reaction network from the *iSB619 S. aureus *model, in principle our tools can produce metabolic reaction networks for any organism in the SEED, by reusing components from the database of coherent reaction subnetworks (Fig. 1B). To test this hypothesis, we have quantified the extent to which the metabolic reaction networks of three other published genome-scale metabolic models (the *iJR904 E. coli *model [24], the *iIT341 H. pylori *model [27], and the *Oliveira, et al., L. lactis *model [28]) can be assembled from coherent reaction subnetworks already in the database, based solely on their genome annotations in the 65 subsystems used to assemble the *S. aureus *metabolic reaction network.

**Figure 6:**
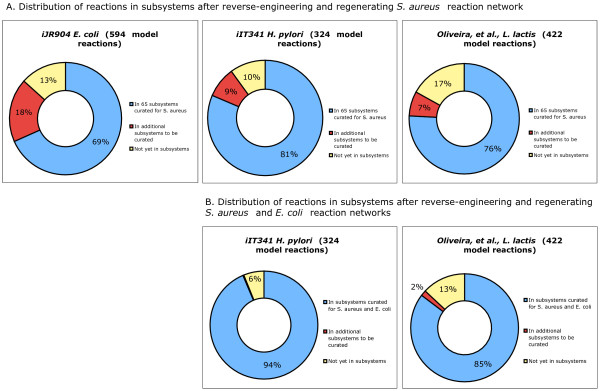
**Cumulative effect of the subsystems approach to model generation**. (A) Reverse-engineering the reaction network from the *S. aureus *model into the SEED establishes a database of curated reactions and scenarios that serves as a platform for reaction network generation for other genomes. The pie graphs depict the percentage of the total number of reactions in three existing models that are covered by the current SEED database and the percentage that remains to be curated. (B) The graphs for the *H. pylori *and *L. lactis *models assume the curation of both the *S. aureus *and the *E. coli *models (see text for details). Data are derived from Additional Files 1 and 3.

We found that in all three cases, a large percentage of the model reactions are already accounted for by the reactions associated with functional roles in the 65 subsystems: 406 out of 594 reactions (~69%) in the case of the *iJR904 E. coli *model, 264 out of 324 reactions (~81%) in the case of the *iIT341 H. pylori *model, and 320 out of 422 reactions (~76%) in the case of the *Oliveira, et al., L. lactis *model (Fig. 6A and Additional File 1). In addition, we found that our database already contains coherent reaction subnetworks with paths through many of the scenarios for the three organisms in these subsystems: 111 scenarios for *E. coli*, 40 scenarios for *H. pylori*, and 74 scenarios for *L. lactis *(Table 3 and Additional File 2). The majority of the reactions in these models that were not mapped to functional roles in the 65 subsystems were automatically associated with functional roles in additional subsystems by the reverse-engineering process (see Methods). For example, some of the reactions in the *iJR904 E. coli *model were mapped to functional roles in two subsystems related to cell wall synthesis: *KDO2 Lipid A Biosynthesis *and *LOS Core Oligosaccharide Synthesis*. These subsystems were not needed for modeling *S. aureus*, which is a gram-positive bacterium. Once we have applied our tools to create coherent reaction subnetworks in these additional subsystems, we will have accounted for another 108 reactions (for a total of ~87%) in the case of the *iJR904 E. coli *model, another 28 reactions (for a total of ~90%) in the case of the *iIT341 H. pylori *model, and another 30 reactions (for a total of ~83%) in the case of the *Oliveira, et al., L. lactis *model (Fig. 6A). The remaining reactions in these models which were not mapped to functional roles by the reverse-engineering process will require more effort, to determine if they can be mapped to existing functional roles in subsystems, or whether new functional roles and/or subsystems are needed to account for them.

**Table 3:** Capitalizing on common aspects of metabolism: reuse of scenarios

**Category**	**Subsystems**	**Scenarios**	***S. aureus***^a^	***E. coli***^a^	***H. pylori***^a^	***L. lactis***^a^
Amino Acids	23	34	34	25	10	15
Carbohydrates	15	39	36	35	6	23
Cell Wall	3	8	8	6	4	7
Lipids	3	9	9	9	2	1
Nitrogen Metabolism	1	1	1	1	0	0
Nucleotide Metabolism	6	22	22	21	14	19
One Carbon	2	5	5	3	1	3
Redox	5	3	3	3	1	1
Sulfur	1	1	1	1	0	0
Vitamins and Cofactors	6	11	9	7	1	5

**Totals**	65	133	128	111	40	74

a. Numbers represent scenarios implemented by the organism-specific reaction network in each category of metabolism.

The *iJR904 E. coli *model is the most complete genome-scale metabolic model published to date, and we are currently applying our tools to regenerate the full reaction network in the SEED. To demonstrate further the cumulative effect of our approach to reaction network generation, we have compared the reactions from the three additional models that were not mapped to functional roles in the 65 subsystems, and determined the overlapping set of reactions from the *H. pylori *and *L. lactis *models that will be mapped to functional roles in other subsystems in the process of regenerating the *E. coli *model in the SEED. In the case of the *iIT341 H. pylori *model, there will only be 20 remaining reactions to map to functional roles in subsystems. In the case of the *Oliveira, et al., L. lactis *model, there will only be 55 remaining reactions to map to functional roles (Fig. 6B and Additional File 3).

## Discussion

Although our work to date has focused on regenerating reaction networks from published genome-scale metabolic models, our tools can be used to generate a preliminary reaction network for any organism in the SEED based solely on its genome annotation (Fig. 1B). As with other methods for generating genome-scale metabolic reaction networks [12-15,18,19], the process of refining the reaction network until it is complete and coherent requires manual effort. The amount of manual effort required depends upon the quality of the preliminary reaction network, which in turn depends upon the quality of the annotation. An underlying goal behind our suite of tools is to narrow the gap between the preliminary reaction network and the complete and coherent reaction network as much as possible, so as to minimize the amount of manual effort required. We anticipate that the database of coherent reaction subnetworks will enable the generation of substantially complete and coherent preliminary reaction networks, focusing manual efforts on resolving gaps that are identified by the path-finding tool (Fig. 4B), and on creating new reaction subnetworks for areas of metabolism not yet represented in the database.

The collection of transport and biomass information from the published physiological literature and addition of corresponding reactions to the reaction network requires a significant manual effort for each organism, and is currently a bottleneck in generating a reaction network suitable for flux balance analysis. To date, this problem has not been adequately addressed in systems designed to facilitate genome-scale reaction network reconstruction [12-15,18,19], nor have we addressed this problem with our current set of tools. However, it may be possible to hypothesize computationally which transport reactions an organism uses, and which biomass components an organism synthesizes, based on the existence of paths through scenarios that represent catabolic pathways for common substrates, or anabolic pathways for common biomass components.

Our approach to automated genome-scale metabolic reaction network generation represents two important advances when compared to previously published methods [12-15,18,19]. The first advance is a process for verifying the completeness and coherence of an overall reaction network by constructing a database of coherent reaction subnetworks that represent interconnected metabolic components. In contrast, most published methods for network generation do not explicitly provide support for the verification process. IdentiCS [15] and metaSHARK [14] provide automated annotation, assembly and visualization of a preliminary reaction network. The GEM System [12] additionally provides a heuristic for filling gaps in sets of consecutive reactions. However, these three systems do not provide tools for iterative refinement and verification of the preliminary network. The Pathway Tools software [13] takes an existing annotation and produces a pathway/genome database (PGDB), which includes predicted metabolic pathways and associated reactions for an organism. The software enables the visualization and manual refinement of the preliminary reaction network, but does not include tools for verification of the network. Segrè *et al. *[19] describe a process that builds upon Pathway Tools and includes an algorithm for the verification of the completeness and coherence of the overall reaction network, but do not describe a process for resolving gaps that are identified in the network. As discussed above, our approach enables resolving such gaps during the process of creating and assembling coherent reaction subnetworks (Fig. 1B). The AUTOGRAPH-method [18] is unique among previously published methods in that it incorporates information from published genome-scale reaction networks to produce a preliminary reaction network for a particular organism. However, no process for verifying and refining the preliminary network is described.

The second advance is the tight integration of our approach and our tools with a community-based genome annotation and analysis tool. From its inception, the SEED was designed to serve as a repository and clearinghouse for parallel annotation projects across all sequenced genomes. Because of the tight integration of our approach with the SEED, our tools can be used at all stages of every genome annotation project in the SEED, and reaction subnetworks created for one project are immediately available for all the other projects. This is in contrast with, *e.g.*, the Pathway Tools software [13], which is downloaded and installed locally by each genome annotation project, and creates a standalone PGDB for each project. Likewise, the published methods discussed above all focus on creating a reaction network for a single organism and, with the exception of the AUTOGRAPH-method, do not describe a process for reusing components of reaction networks previously developed for other organisms.

## Conclusion

We have described a method for automating the generation of substantially complete and coherent genome-scale metabolic reaction networks from annotated genomes. Our method builds on the subsystems approach to genome annotation and analysis embodied in the SEED. The SEED already provides well-curated genome annotations for central and intermediary metabolism across many organisms. We have extended the SEED by curating associations between reactions and functional roles in subsystems based on metabolic context. We have created tools for encoding components of reaction networks in subsystems, and verifying their coherence and interconnections. We have created tools for assembling these components into organism-specific complete and coherent reaction networks. We have demonstrated that our process can regenerate the reaction network from a published genome-scale metabolic model, and that it produces a cumulative effect supporting the subsequent generation of other reaction networks from published models. Our future work will focus on applying this process to generating reaction networks for new organisms, eventually extending to all organisms annotated in the SEED, thus producing a repository of organism-specific complete and coherent reaction networks. We envision that this repository will be useful for interpretation of large-scale data sets generated for metabolic genomics [38].

## Methods

### Reverse-engineering existing genome-scale metabolic models into the SEED

Each of the four genome-scale metabolic models [20,24,27,28] that we reverse-engineered contains a list of compounds, a list of reactions, and a list of gene-reaction associations. The list of reactions can be broken into three categories: (1) reactions that take place within the cell, (2) transport reactions, which move compounds into and out of the cell, and (3) exchange reactions, which simulate compounds entering and leaving the extra-cellular space. Our reverse-engineering process focused on mapping the first category of reactions to KEGG reactions, and associating these with functional roles in subsystems.

Our reverse-engineering process started with mapping the compounds in the existing models to compounds in KEGG. Much of this could be done automatically, since the models for the most part use standard compound names and abbreviations that are also used by KEGG. In some cases, a model compound may map to more than one KEGG compound id, *e.g., D-Glucose *can be mapped to C00031 (also *D-Glucose*), as well as C00221 (*beta-D-Glucose*) and C00267 (*alpha-D-Glucose*). We retained all possible mappings because different KEGG reactions use different compound ids. The next step was to map model reactions to KEGG reactions. Our criterion was a perfect match on all substrates and products, except for protons, since the models and KEGG sometimes account for them differently (*e.g., nitrite *in the models is *NO*_2_, whereas in KEGG it is *HNO*_2_). Again, much of this could be done automatically. For model reactions that did not perfectly match a KEGG reaction, we searched by hand for similar KEGG reactions, looking for cofactor differences, equivalent combinations of reactions, and so on. In these cases we accepted an inexact match, but noted the discrepancy. In a few cases we were unable to map model reactions to any KEGG reaction. For example, the models do not contain reactions representing the individual steps in the synthesis of specific fatty acids, but instead represent the cumulative synthesis of specific fatty acids as one "reaction". In these cases we did not process the reactions any further, but noted that they should be added "as is" to the organism's metabolic reaction network. This required the specification of several more reactions to convert between KEGG compound ids and the model compound ids in these added reactions. Additional Files 4 and 5 document the mapping from compounds and reactions in the *iSB619 S. aureus *model to KEGG compounds and reactions.

When mapping model reactions from the *iSB619 S. aureus *model to KEGG reactions, we noted that approximately 40% of the reactions had different reversibilities between these two sources (*e.g.*, a reaction asserted to be one-way in the model is represented as bidirectional in KEGG). Some of these discrepancies are clearly errors in the KEGG database (*e.g.*, the KEGG pathway map agrees with the model whereas the KEGG flat file does not); we have brought these to the attention of the KEGG database curators, and they have been fixed. Efforts are underway to correct and improve upon the compound and reaction information in KEGG (*e.g.*, [39]). In light of this, we have structured our database so that we have the option of using reaction information from sources other than KEGG.

Once we completed mapping the model reactions to KEGG reactions, we used the gene-reaction associations from each of the models to associate KEGG reactions with functional roles in SEED subsystems. For each gene in a model, we identified all subsystems (if any) where the gene is annotated to a functional role. Most of these functional roles specify an EC number; in some cases, they have also been assigned KEGG reactions by the original subsystem author. In addition, we retrieved the KEGG reaction(s) annotated to the gene from the KEGG GENE database. Thus, for each model, we collected up to three lines of evidence for determining whether to assign a given KEGG reaction to a functional role: the gene-reaction association and corresponding KEGG reaction from a model; KEGG's gene-reaction association for the organism; and the functional role's EC number and KEGG reaction. We recorded this evidence in the SEED database for subsequent manual curation. In addition, if all three lines of evidence matched, we automatically assigned the KEGG reaction to the functional role, and noted in the SEED database that an automatic assignment was made. At the end of this process we had a preliminary set of 805 associations between KEGG reactions and functional roles in 205 subsystems, with links back to the gene-reaction associations from the four models.

### Manual curation of associations between functional roles and reactions

After reverse-engineering the four models into the SEED, we manually curated associations between functional roles and reactions by checking every automatic assignment of reactions and all links to reaction evidence. When the three lines of evidence for assigning reactions to functional roles were inconclusive, we examined the evidence in the context of the appropriate KEGG pathway to make a determination. For example, in the *Embden-Meyerhof and Gluconeogenesis *subsystem, the subsystem author assigned reaction R00299 (*ATP + D-Glucose *<=> *ADP + D-Glucose 6-phosphate*) to the *Glucokinase (EC 2.7.1.2) *functional role. One of the models, *L. lactis*, matched this reaction, but the other three models and KEGG all matched R01600 (*ATP + beta-D-Glucose *<=> *ADP + beta-D-Glucose 6-phosphate*). After consulting the *Glycolysis*/*Gluconeogenesis *map in KEGG, which represents the catabolism of both the alpha- and beta- forms of glucose, we decided that it was appropriate to assign both reactions to the functional role, as well as R01786 (*ATP + alpha-D-Glucose *<=> *ADP + alpha-D-Glucose 6-phosphate*), so that all three forms of glucose would be represented in the reaction network generated from this subsystem. In some cases, we added reactions to functional roles based on evidence from the functional roles and the KEGG pathways alone, such as R01450 for *Lactate racemase (EC 5.1.2.1)*, which is in the *Fermentations: Lactate *subsystem and the *Pyruvate Metabolism *KEGG pathway, but not associated with a gene in any of the four models (it is in the *iIT341 *model, without a gene-reaction association).

### Availability of code and database

We are currently working with the SEED development group to incorporate our extensions to the SEED into the standard open-source SEED distribution, available at [30]. Our associations between reactions and functional roles have already been added to subsystems in this distribution. We anticipate that our tools will be available through the SEED in May of 2007.

## Authors' contributions

AB and MD conceived and supervised the project, and drafted the manuscript. MD and KF developed the software. JG and PB contributed to the software, and MR contributed to data analysis. All authors read and approved the final manuscript.

## Supplementary Material

Additional File 1 **Reverse-engineering reactions from *E. coli*, *H. pylori*, and *L. lactis *models after *S. aureus *network curation**. The table shows the distribution of reactions in relation to subsystems and functional roles in the SEED subsequent to *S. aureus *curation. Click here for file

Additional File 2 **Subsystems and scenarios used in reaction network generation**. The table lists the subsystem names, subsystem abbreviations, and scenario names that were curated in preparation for reaction network generation; subsystems are grouped according to metabolic categories. An "x" indicates those subsystems and scenarios used in the generation of the *S. aureus iSB619 *reaction network. Subsystems and scenarios identified as complete for generation of *E. coli, H. pylori *and *L. lactis *networks are also indicated. Click here for file

Additional File 3 **Reverse-engineering reactions from *H. pylori *and *L. lactis *models after *S. aureus *and *E. coli *network curation**. The table shows the distribution of reactions in relation to subsystems and functional roles in the SEED subsequent to *S. aureus *and *E. coli *curation. Click here for file

Additional File 4 **Distribution of reverse-engineered reactions from *S. aureus iSB619 *model**. The table shows the distribution of reactions in relation to subsystems and functional roles in the SEED for the *S. aureus *model. Click here for file

Additional File 5 **Correspondences among *iSB619 *model and KEGG compound and reaction identifiers**. The tables provide correspondences among *iSB619 *model compound and reaction identifiers and KEGG compound and reaction identifiers. Model reactions without matches to KEGG reactions or with inexact matches to KEGG reactions used in the generation of the *S. aureus *model are indicated by "add as is" and "...is similar", respectively. Conversion reactions are also listed. Click here for file
