# Supplementary material for: Toward the automated generation of genome-scale metabolic networks in the SEED
Source: BMC Bioinformatics. 2007 Apr 26;8:139. doi: 10.1186/1471-2105-8-139 (PMC1868769; doi:10.1186/1471-2105-8-139)
Supplement: Additional File 1 — Reverse-engineering reactions from E. coli, H. pylori, and L. lactis models after S. aureus network curation. The table shows the distribution of reactions in relation to subsystems and functional roles in the SEED subsequent to S. aureus curation. [file 1471-2105-8-139-S1.pdf]

Reverse-engineering reactions from *E. coli*, *H. pylori*, and *L. lactis* models after *S. aureus* model curation

			E. coli	*H. pylori*	*L. lactis*
Reactions to Reverse Engineer	Total Number of Reactions[a]		648	357	467
			Reaction Counts		
	Reactions to Add "As is"[b]		54	33	45
	Reactions to Map to Functional Roles	In Curated Subsystems[c]	406	264	320
		In Additional Subsystems[d]	108	28	30
		Remaining Reactions[e]	80	32	72

a. Reaction total does not include transport and exchange reactions, and "dead end" reactions (*i.e.*, reactions whose substrates are never produced or whose products are never consumed) specified by the published models.

b. Reactions in respective models that combine many reactions into one for synthesis of organism-specific properties (e.g., fatty acid synthesis).

c. Reactions in the models that map to functional roles in the 65 subsystems curated for *S. aureus* network generation.

d. Reactions in the models that map to functional roles in existing subsystems that were not curated in association with *S. aureus* network generation.

e. Reactions not yet associated with functional roles in subsystems.
