# Supplementary material for: Toward the automated generation of genome-scale metabolic networks in the SEED
Source: BMC Bioinformatics. 2007 Apr 26;8:139. doi: 10.1186/1471-2105-8-139 (PMC1868769; doi:10.1186/1471-2105-8-139)
Supplement: Additional File 2 — Subsystems and scenarios used in reaction network generation. The table lists the subsystem names, subsystem abbreviations, and scenario names that were curated in preparation for reaction network generation; subsystems are grouped according to metabolic categories. An "x" indicates those subsystems and scenarios used in the generation of the S. aureus iSB619 reaction network. Subsystems and scenarios identified as complete for generation of E. coli, H. pylori and L. lactis networks are also indicated. [file 1471-2105-8-139-S2.pdf]

Subsystems and scenarios used in reaction network generation

Category	Subsystem	Abbreviation	Scenario	*S. aureus*	*E. coli*	*H. pylori*	*L. lactis*
AminoAcids	Ammonia assimilation	NH3 assim	NH3 to Glutamine	x	x		
	Arginine Biosynthesis	Arginine	Arginine synthesis	x	x		
	Arginine Putrescine and 4-aminobutyrate degradation	ArgPut deg	Arginine to Proline	x			
			Arginine to Putrescine	x	x		
	Branched-Chain Amino Acid Biosynthesis	Branched AA	Isoleucine Leucine Valine synthesis	x	x		x
			Oxoisovalerate generation	x	x		x
	Chorismate Synthesis	Chorismate	Chorismate synthesis	x	x	x	x
	Glutamate, aspartate and asparagine biosynthesis	GluAspAsn	Asparagine synthesis	x	x		x
			Aspartate synthesis	x	x		
			Glutamate racemase	x	x	x	x
			Glutamate synthesis	x	x	x	
			Glutamine synthesis	x	x		x
	Histidine Biosynthesis	Histidine	Histidine synthesis	x	x		x
	Histidine Degradation	Hist deg	Histidine to Glutamate	x			
	Isoleucine degradation	Isoleu deg	Isoleucine to 2MethylbutyrylCoA	x			
			Isoleucine to PropanoylCoA	x			
	Leucine Degradation and HMG-CoA Metabolism	Leucine deg	Leucine to IsovalerylCoA	x			
	Lysine Biosynthesis DAP Pathway	Lysine	LL 2 6 Diaminoheptanedioate generation	x	x	x	x
			Lysine synthesis	x	x	x	
	Methionine Biosynthesis	Methionine	Methionine synthesis from homoserine	x	x		
			Methionine to Adenosyl Methionine	x	x	x	x
	Methionine Salvage	MethSalvage	*incomplete scenario*				
	Phenylalanine synthesis	Phenylalanine	Phenylalanine synthesis	x	x		
	Polyamine Metabolism	Polyamine	Putrescine to Spermine	x			
	Pyruvate Alanine Serine Interconversions	PyrAlaSer	Alanine Synthesis	x			
	Serine Biosynthesis	Serine	Glycine synthesis	x	x	x	x
			Serine synthesis	x	x		
	Threonine degradation	Threon deg	Threonine to Glycine and Acetaldehyde	x	x		
			Threonine to Oxobutanoate	x			x
	Threonine synthesis	Threonine	Homoserine generation	x	x	x	x
			Threonine synthesis	x	x	x	x
	Tryptophan synthesis	Tryptophan	Tryptophan synthesis	x	x	x	x
	Tyrosine synthesis	Tyrosine	Tyrosine synthesis	x	x		
	Valine degradation	Valine deg	Valine to IsobutyrylCoA	x			
	cysteine biosynthesis	cysteine	Cysteine synthesis	x	x		x
Carbohydrates	Embden-Meyerhof and Gluconeogenesis	EMP	Fructose16BP entry point	x	x		x
			Fructose6P entry point	x	x		x
			GAP entry point	x	x		x
			Gluconeogenesis		x	x	
			Glucose6P generation	x	x	x	x
			Glycerate3P generation	x	x		x
			GlyceroneP entry point	x	x		x
			GlyceroneP generation	x	x		x
			Glycolysis	x	x		x
			PEP generation	x	x		x
	Entner-Doudoroff Pathway	EntnerDoff	Gluconate to PhosphoDGluconate	x	x		x
	Fermentations: Lactate	FermLac	Bifidium precursors				x
			Heterofermentation precursors				x
			Homofermentation	x	x		x
	Fermentations: Mixed acid	FermMixed	PEP to Formate	x	x		
	Fructose utilization	Fructose	Fructose16BP generation	x	x		x
	Galactose degradation	Lactose	Lactose6P utilization	x			
	Glycerol and Glycerol-3-phosphate Uptake and Utilization	Glycerol	Glycerol3P and Glycerol to GlyceroneP	x	x		x
	Mannitol Utilization	Mannitol	Fructose6P generation	x	x		x
	Mannose Metabolism	Mannose	Fructose6P generation	x	x		x
	Pentose phosphate pathway	PPP	Erythrose4P generation	x	x		
			Glycolysis bypass	x	x		
			PRPP generation	x	x		x
			PhosphoDGluconate entry point	x	x		x
			Xylulose5P generation	x	x		x
	Pyruvate metabolism I: anaplerotic reactions, PEP	PyrMetab 1	PEP generation	x	x		
			PEP to TCA metabolites	x	x		
			Pyruvate generation	x	x		
			Pyruvate to TCA metabolites	x	x		
	Pyruvate metabolism II: acetyl-CoA, acetogenesis from pyruvat	PyrMetab II	Acetate to Acetaldehyde	x	x		x
			AcetylCoA generation	x	x		x
	Sucrose Metabolism	Sucrose	Sucrose6P and Glucose to Glucose6P	x	x	x	x
			UDPglucose generation	x	x		
	TCA Cycle	TCA	Fumarate entry point	x	x	x	
			Oxaloacetate to Succinate	x	x	x	
			Oxoglutarate generation	x		x	
			SuccinylCoA entry point	x	x		
			TCA cycle	x	x		
	Trehalose Uptake and Utilization	Trehalose	Trehalose6P to Glucose and Glucose6P	x	x		
Cell Wall	Peptidoglycan Biosynthesis	Peptidogly	UDP N acetyl muramate generation	x	x	x	x
	Sialic Acid Metabolism	Aminosugars	D glucosamine1P	x	x	x	x
			N acetyl D glucosamine1P generation	x	x	x	x
			UDP N acetyl D galactosamine generation	x	x	x	x
			UDP N acetyl D glucosamine generation	x	x	x	x
			UDP N acetyl D mannosamine generation	x	x		x
	Teichoic and lipoteichoic acids biosynthesis	(Lipo)teich	CDPglycerol generation	x			x
			Diglucosyl diacylglycerol generation	x			
Lipids	Fatty Acid Biosynthesis FASII	FattySynth	Malonyl CoA generation	x	x	x	
	Glycerolipid and glycerphospholipid metabolism	Glyc(P)lipids	CDP diacylglycerol generation	x	x		
			Cardiolipin synthesis	x	x		
			Diacylglycerol synthesis	x	x		
			For 3 D Glucosyl 1 2 diacylglycerol generation	x	x	x	x
			Phosphatidylethanolamine synthesis	x	x		
			Phosphatidylglycerol generation	x	x		
			Phosphatidylserine synthesis	x	x		
	fatty acid metabolism	FattyMetab	Hexadecanoate to AcetylCoA	x	x		
Nitrogen Metabolism	Nitrate and nitrite ammonification	Nitra/ite	Nitrite to Ammonia reduction	x	x		

Category	Subsystem	Abbreviation	Scenario	*S. aureus*	*E. coli*	*H. pylori*	*L. lactis*
Nucleotide metabolism	De Novo Purine Biosynthesis	IMP	AICAR to IMP	x	x		x
			IMP synthesis	x	x		x
	De Novo Pyrimidine Synthesis	UMP	UMP synthesis from CarbamoylP	x	x	x	x
			UMP synthesis from Glutamine	x	x	x	x
	Purine conversions	Purines	ADP generation	x	x	x	x
			AMP generation	x	x	x	x
			Adenine to AMP	x	x	x	x
			AdenosinePP to AMP	x	x	x	x
			GDP generation	x	x	x	x
			GMP generation	x	x	x	x
			GTP generation	x	x	x	x
			dAMP generation	x	x	x	x
			dGMP generation	x	x	x	x
	Ribonucleotide reduction	RiboReduc	dADP generation	x	x	x	x
			dCTP generation	x	x		x
			dGDP generation	x	x	x	x
	Ribose and deoxyribose phosphate metabolism	(Deoxy)Rib	Deoxyribose1P generation	x	x		x
			Ribose5P generation	x	x		x
	pyrimidine conversions	Pyrimidines	CMP generation	x	x		
			CTP generation	x	x	x	
			dCMP generation	x			
			dTMP generation	x	x		x
One Carbon	Glycine cleavage system	Gly cleavage	Glycine cleavage	x	x		
	One-carbon metabolism by tetrahydropterines	One carbon	Formyltetrahydrofolate generation	x			x
			Methylenetetrahydrofolate generation	x			x
			Methyltetrahydrofolate generation	x	x		x
			Tetrahydrofolate generation	x	x	x	
Redox	Dehydrogenase complexes	Dehydro	*no scenario*				
	F0F1 type ATP synthase	ATP	*no scenario*				
	Formate hydrogenase	Formate	Formate dehydrogenase	x	x		
	Respiratory dehydrogenases 1	RespDH	NADH ubiquinone oxidation	x	x		x
	Succinate dehydrogenase	FAD dehydro	FADH2 ubiquinone oxidation	x	x	x	
Sulfur	Inorganic Sulfur Assimilation	Sulfide	Sulfate to Sulfide	x	x		
Vitamins and Cofactors	Coenzyme A Biosynthesis	CoA	CoA synthesis	x	x	x	
	FMN and FAD biosynthesis	FMN/FAD	FAD synthesis	x			
	Folate Biosynthesis	Folate	Dihydrofolate to Tetrahydrofolate	x	x		x
			Tetrahydrofolate generation	x			x
	Isoprenoid Biosynthesis	FarnesylPP	DOXP pathway		x		
			Mevalonate pathway	x			x
	NAD and NADP cofactor biosynthesis global	NAD/NADP	De novo from Trp and Asp		x		
			Nicotinamide to NAD	x	x		x
			Nicotinamide to NADP	x	x		x
	Porphyrin, Heme, and Siroheme Biosynthesis	Heme	Heme synthesis	x			
			Siroheme synthesis	x	x		
