# Supplementary material for: Toward the automated generation of genome-scale metabolic networks in the SEED
Source: BMC Bioinformatics. 2007 Apr 26;8:139. doi: 10.1186/1471-2105-8-139 (PMC1868769; doi:10.1186/1471-2105-8-139)
Supplement: Additional File 4 — Distribution of reverse-engineered reactions from S. aureus iSB619 model. The table shows the distribution of reactions in relation to subsystems and functional roles in the SEED for the S. aureus model. [file 1471-2105-8-139-S4.pdf]

Distribution of reverse-engineered reactions from *S. aureus* iSB619 model

Reactions to Reverse Engineer	Total Number of Reactions[a]		427
			Reaction Counts
	Reactions Added "As is"[b]		33
	Reactions Mapped to Functional Roles	Exact match to KEGG reaction	365
		Inexact match to KEGG reaction[c]	29

a. Reaction total does not include transport and exchange reactions, and "dead end" reactions (*i.e.*, reactions whose substrates are never produced or whose products are never consumed) specified by the published model.

b. Reactions in *iSB619* model that combine many reactions into one for synthesis of *S. aureus* specific properties (*e.g.*, fatty acid synthesis).

c. Reactions in the model that differ slightly from the KEGG reaction database (*e.g.*, cofactor usage).
